# Barbie’s new look: Exploring cognitive body representation among female children and adolescents

**DOI:** 10.1371/journal.pone.0218315

**Published:** 2019-06-25

**Authors:** Amy Nesbitt, Catherine M. Sabiston, Melissa deJonge, Shauna Solomon-Krakus, Timothy N. Welsh

**Affiliations:** Faculty of Kinesiology and Physical Education, University of Toronto, Toronto, ON, Canada; Universita degli Studi di Udine, ITALY

## Abstract

The original Barbie doll’s unrealistic body shape can negatively affect young girls’ body image. Mattel produced new Barbie dolls with “tall”, “curvy”, and “petite” body types, yet how girls perceive and evaluate the three new Barbie body types remains unknown. This study investigated whether young girls engage in an automatic “self-other matching” process when viewing the different Barbie doll representations. Female children and adolescents (*N* = 38; *M*_age_ = 10; 6–14 years old; *SD* = 2.24 years) completed a body-part compatibility task to provide an index of how they implicitly relate cognitive representations of their own body to the different doll images. Significant (*p <* .05) body-part compatibility effects emerged for the original, curvy and petite dolls, but not for the tall Barbie. These findings indicate that girls engage in a self-other body matching process when viewing Barbie, but that the strength of this matching is influenced by the doll’s body type. Results provide new evidence on the underlying cognitive mechanisms that occur when girls are exposed to physique-salient toys, and may have implications for young girls’ body image development and use of appearance-based social comparisons.

## Introduction

Playtime is a critical aspect of childhood development [1] because learning occurs by modelling the behaviors and internalizing the ideals and values of other people who are engaging in social interactions [2,3]. For generations of young girls, the Barbie doll has been a popular toy of choice. These dolls, however, have been critiqued for promoting unhealthy thinness and appearance-based ideals [4]. That is, Barbie is thought to represent the longstanding idealized female figure of beauty, and her body proportions have been identified as both unrealistic and unhealthy in the popular press and empirical papers [5,6]. Though not all authors agree on Barbie’s negative impact [7,8], Barbie dolls have been criticized for providing young girls with a tangible physique-salient representation of unrealistic female body shapes. In fact, there are well-documented negative body image effects related to exposure to Barbie, including reduced body esteem and body satisfaction, higher body size discrepancy, thin-ideal internalization and desire for thinness, and restrictive eating behavior [9–12]. These results are troubling because reduced body esteem and an increased drive for thinness are indicators of body dissatisfaction. Body dissatisfaction can in turn act as a precursor to mental health outcomes such as clinical depression and eating disorders [13–15].

In an effort to address these issues, Mattel recently introduced three new Barbie dolls with varying body types: “tall”, “curvy”, and “petite” [16,17]. Studies examining how young girls perceive and evaluate the three new Barbie body types have, to our knowledge, yet to be conducted. Furthermore, researchers have commonly focused on young girls’ perceptual, affective and behavioral responses to the original Barbie, while the underlying cognitive processes have yet to be explored. The present study was designed to address these shortcomings and provide new insight into how the new and old (original) Barbie dolls are evaluated, as well as some of the cognitive mechanisms that are engaged when young girls view these dolls.

Cognitive theories, such as social comparison theory [18] and self-schema theory [19], have helped to explain the underlying cognitive mechanisms believed to be involved when humans view physique-salient media. Humans have a strong tendency to compare themselves to other people in an effort to gain an accurate self-evaluation (e.g., in terms of appearance) [18]. According to social comparison theory, individuals tend to draw comparisons to those who are most similar, and will either engage in upward social comparisons (the other’s body is closer to the ideal body compared to the self) or downward comparisons (the other’s body is farther from the ideal body compared to the self) [18]. Exposure to physique-salient media and products are thought to foster upward social comparisons which can lead to negative evaluations of the self and increased body dissatisfaction [18,20,21]. Previous research has shown that such social comparative processing (e.g., upward and downward social comparisons) begins to occur in early childhood [22,23]. Moreover, young girls have demonstrated higher social comparative tendencies and in comparison to males are more affected by the negative consequences of appearance-based social comparisons [14,21]. This increased tendency to engage in social comparisons is of critical concern, given that social comparisons occur through automatic, cognitive processes, which we are believed to have little-to-no control over [24,25].

Self-schema theory adheres to the notion of an automatic self-other comparison system and suggests that individuals possess cognitive-based, internal representations of the self that exist across multiple domains including social roles, physical characteristics, and interests [19]. Previous research has focused on such self-schema constructs to measure core beliefs about the importance of appearance, as well as appearance discrepancies between self-perceived ideal and actual selves [26–30]. More recent research has focused on the self-schema of the body (the “body schema”) to further understand the cognitive processing of body-related information, and the implications of such implicit processes for one’s body image [31]. For the purpose of the present study, body schema is defined as an internal, cognitive representation of the human body which not only enables one to know where their own body is in space, but becomes activated during the observation of another human body form (see Peelen & Downing, 2007 [32] for a review of the neural structures involved in the processing of observed bodies) [31–35]. The body schema is thought to provide information that allows individuals to perceive and act in their sensory and social environment [36].

Activation of the body schema is thought to be a core process leading to a body-mapping effect where the body parts of others are represented with respect to the observers’ own body and is suggested to play a role in social cognition processes such as empathy, imitative behavior, and coding self-produced and observed actions [34,37,38]. In other words, activation of the body schema is suggested to provide a basis for understanding the physical self along with, and in comparison to the bodies of others [39]. While previous research has primarily focused on understanding the self and others through motor-simulation mechanisms, activation of the body schema may also play a role in social cognition and self-related processing [40–42]. Given that individuals are more likely to partake in social comparative processes towards those who are viewed as similar, activation of the body schema may have implications for body image development in young girls.

Indeed, there is neurophysiological evidence suggesting that neural networks involved in the processing of human body-type forms are associated with body image outcomes. Specifically, neural networks (consisting of the lateral fusiform gyrus, inferior parietal cortex and lateral prefrontal cortex) as well as related affective networks (consisting of the anterior cingulate, insula and amygdala) have been highlighted as important brain areas involved in the processing of physique-salient images [43–45]. When exposed to an image of a model portraying the thin ideal, activation of these brain regions has been correlated with the viewer’s level of anxiety [44] and perceptual sensitivity to their own body [34]. In addition, Preston and Ehrsson [43] recently demonstrated that illusory obesity (the multisensory illusion of having an obese body) produced significantly lower body satisfaction compared to pre-illusion baseline scores among a sample of female adults, which was associated with anterior cingulate cortex and anterior insula cortex activation [43]. Further, there is neurophysiological evidence that there is an area of the inferior occipital cortex, the extrastriate body area (EBA), that is specialized for, or becomes most active when viewing, human body forms [32]. Thus, appearance-focused social comparisons likely occur through the automatic activation of these specialized brain areas for the processing of other peoples’ bodies, but with specific reference to the observer’s cognitively-based internal representation of their own body. While it is theorized that the same neurophysiological mechanisms are present in young children and adolescents, experimental studies are required with younger age groups to better understand the development and activation of these brain areas in response to physique-salient media across the life span [46,47]. To date, our understanding of body schema development and activation has relied on behavioural studies of children’s responses to motor or visual stimuli [46,47] and of studies with adults.

The cognitive activation of the body schema has been examined in behavioural studies utilizing the body-part compatibility task [48]. During this task, participants are presented with an image of a human body (or a human body form) with either a red or blue target stimulus appearing on the model’s hand or foot. Participants are instructed to execute a hand-response for a red stimulus and a foot-response for a blue stimulus, regardless of where it is presented on the body. In these studies, a pattern of responses times (RTs), termed the body-part compatibility effect, emerges. The body-part compatibility effect is defined as a pattern of RTs in which RTs are shorter for body-part compatible trials, in which the responding limb and location of the color stimulus on the model is the same (e.g., red target on the hand in the image), compared to body-part incompatible trials, in which the responding limb and location of the color stimulus on the model is different (e.g., red target on the foot in the image). Body-part compatibility effects are thought to occur because exposure to the human body or human body form subsequently activates the participant’s cognitive body schema [34,35,48]. The presentation of a color stimulus on the model’s hand or foot primes or further excites that specific body part within the viewer’s body schema. This body-part priming in the body schema is thought to facilitate faster RTs when the response is made with the limb that is the same as the excited body part, whereas interference and longer RTs may arise when the participant has to execute a response with a different, non-primed/excited body part [48]. Thus, the presence of the body-part compatibility effect is thought to reflect activation of the body schema and related self-other matching during the observation of another individual’s body.

In support of the notion that the body-part compatibility effect indexes body-schema activation and the self-other body matching mechanisms that may be involved in social comparisons, the presence and magnitude of the effect has been shown to be determined by the species and posture of the model in the image [35,49], the tendency for the observer to engage in social comparison [31], and the age and gender of the observer and model [50]. While most of the body-part compatibility work to date has focused on adult participants, Pacione et al. [50] asked male and female children and adolescents to complete a body-part compatibility effect task when viewing images of 7, 11, and 15 years old males [50]. The authors found a peer group effect in that the male participants aged 10–12 and aged 13–16 years old showed body-part compatibility effects only when they viewed the images of the 11 year old and 15 year old male, respectively. None of the female participants or the males aged 7–9 years demonstrated body-part compatibility effects with any of the male images, regardless of the age of the model. This peer group effect for the males aged 10–12 and 13–16 years was suggested to occur because these males are more likely to engage in play, social interactions, and social comparisons with males of a similar age. A similar explanation was provided for the absence of a body-part compatibility effect for the females in the study–only male images were used and the female participants were unlikely to have engaged in consistent self-other matching and social comparisons with males. Finally, it is possible that the males aged 7–9 years did not demonstrate a body-part compatibility effect because this process is not sufficiently developed in males at this age. Overall, these peer group findings demonstrate the sensitivity of the body-part compatibility effect to the relative characteristics of the observer and the model and, methodologically speaking, the use of the effects as an index of self-other matching in children and adolescents.

In the present study, the presence or absence of the body-part compatibility effect was used to index the body schema activation and social self-other comparisons that may occur when young females view images of the different Barbie dolls. Young females aged 6 to 14 years were involved in the current study because an individual’s body schema matures throughout childhood and adolescence, and is influenced by interactions with the environment and other people [36]. Specifically, the body schema develops based on the sensory information provided by the moving body and socially formed images of the body (e.g., peer interactions, family interactions, magazine and television exposure) [28,36]. As a result, the body-part compatibility effect may not emerge for every viewed body for people of every age and, as mentioned, may be influenced by the individuals’ tendency to engage in social comparison as well as the characteristics of the body that is observed [31,50]. For example, in their recent investigation, Pila et al. [31] utilized the body-part compatibility task to test the automatic and involuntary nature of appearance-based social comparisons among young adult women (18–25 years old) during exposure to physique-salient media images–female models with ideal, average or above average body sizes wearing bikinis [31]. In their results, the magnitude of the body-part compatibility effect was moderated by the body type of the model in the image and the participants’ tendencies for social comparison. Few experimental studies have examined the cognitive processing of body-related information during media exposure among youth. As reviewed earlier, however, the results of a recent study suggest that body-part compatibility effects emerge around the age of 10 years, but only for bodies of children of the same gender and of similar ages [50]. These findings suggest that children are able to engage in self-other matching, but only for socially-relevant stimuli perceived as similar to themselves.

Despite these advancements in body schema research, how physique-salient images are processed and subsequently how they influence representations of the body is largely understudied. Particularly, the automatic nature of social comparative processes following physique-salient media exposure among female youth has not been examined. Thus, the present study utilized a novel adaptation of the body-part compatibility task to investigate the cognitive mechanisms through which young girls implicitly engage in appearance-based social comparisons during exposure to various Barbie doll body types. To this end, female children and young adolescent participants completed a body-part compatibility task with images of the different Barbies (i.e., original, tall, petite, and curvy). If body-part compatibility effects emerge with any specific Barbie doll, such a finding would suggest that young girls view the specific Barbie body types as self-relevant, and engage in self-other matching and social comparison processes. These findings may also contribute to our understanding of how young girls perceive and evaluate the new body types of Barbie, which is presently unknown in the empirical literature. Additionally, this line of research may improve our understanding of body schema activation among young girls and the automaticity of social comparative tendencies which appear to drive body dissatisfaction among young girls following Barbie exposure [10]. In other words, the previously reported negative body image effects related to the original Barbie doll may, in part, arise through young girls’ implicit tendency to engage in upward social comparisons.

### Objectives and hypotheses

Although body-part compatibility effects have been observed among older adolescents and young adults [48,50], the effects are relatively understudied among children and younger adolescents [31,35]. Further, it is unknown how and if these social comparison processes are activated when these females view any of the Barbie dolls with different body types. As such, the current study had two main objectives: i) investigate the extent to which young girls engage in self-other matching with the four different Barbie representations (i.e., original, tall, curvy, and petite) and ii) gain an understanding of the age-related development of the self-other matching process in young female children and adolescents. To achieve each of these objectives, the present study was designed to determine whether the body-part compatibility effect emerges when young girls view images of Barbie with different body types, and whether age moderates any effects. Guided by social comparison [18] and self-schema [19] theories, and based on recent evidence for the implicit processing of physique-salient images [31,50], it was hypothesized that the body-part compatibility effect (observed by faster RTs for body-part compatible trials than on incompatible trials) would emerge when participants were exposed to images of the “petite” Barbie (Hypothesis 1). Although none of the Barbie doll proportions are considered child-like, the short stature of the petite Barbie doll, in comparison to the other Barbie dolls, more closely resembles the body type of a female child and/or adolescent, and may be perceived by participants as the most self-relevant. Indeed, if Petite Barbie was scaled to human size, she would be 4”11 with a waist-to-hip ratio of .73 [51]. Based on the World Health Organization Child Growth Standards [52], a height of 4’11 is appropriate for children and young-adolescents, and research has demonstrated that in comparison to the waist-to-hip ratio of original, tall, and curvy Barbie, the petite Barbie displays a more age appropriate and realistic waist-to-hip ratio (e.g., [53, 54]). Guided by research on body schema development [36] and the empirical findings of Pacione et al. [50], it was also expected that age would moderate body-part compatibility effects, with results being stronger and more consistent in the older age group (10–14 years) compared to the younger age group (6–9 years; Hypothesis 2). The inclusion of female participants with a broad age range was necessary in order to explore the developmental course of body-schema activation from childhood to adolescence.

## Methods

### Participants and procedures

Approval of the study was granted by the University of Toronto Health Sciences Research Ethics Board. A community-based sample of female children and adolescents was identified through a local day camp program. Interested parents were contacted with an introductory letter to explain the general purpose of the study, the study protocol, and the inclusion criteria for participation. The young female participants were kept naïve to the exact study purpose and hypotheses. This intentional non-disclosure was necessary to prevent any conscious or unconscious response biases that may affect the results. Parents and children provided informed consent and assent, respectively, prior to beginning data collection, and received financial compensation in the form of a $10 gift card upon completion of the study. All study procedures were carried out in accordance with the institutional guidelines and regulations for human research.

Participants were scheduled to complete the study if the following inclusion criteria were met: (i) identified as female (ii) English speaking and reading age of 8 years old (or grade 2 level by Canadian elementary school standards); (iii) no motor or cognitive deficits that would impact processing of information or reaction times; (iv) normal to corrected-to-normal eyesight (children with color blindness could not participate); (v) age 6 to 14 years; (vi) no prior clinical diagnosis of an eating disorder or major depressive disorder. Following initial screening, a total of 38 female children completed the study protocol—first completing a body-part compatibility task and then a self-report questionnaire.

In the body-part compatibility task, participants were presented with stimuli on a 23” LCD computer screen, sitting in a chair at a desk approximately 70cm away from the monitor. Stimuli consisted of profile images of original, tall, curvy and petite Barbie dolls. All pictures of the dolls were of a frontal view. For each trial, a single, randomized image of Barbie was presented. Each of the Barbie images was digitally altered to be minimally dressed (wearing a bikini to emphasize body shape), and centered on a white background. All four Barbie dolls had the same skin, hair, and eye color to minimize the effect of other appearance-based characteristics on the participants. A single target stimulus of either a red or blue circle (2.5cm in diameter) was superimposed on each Barbie image over one hand or foot. The red and blue targets were equally presented on the hand and the foot, and on the right and left side of the Barbie doll across the different trials in a random order to minimize attentional shift from the fixation cross. Therefore, a total of 8 images were created for each Barbie body type consisting of the factorial combination of target color (red/blue), location (hand/foot), and target side (right/left).

To perform the task, participants placed their dominant thumb over a button in a plunger held in the dominant hand, and their dominant foot over a pedal. The participants were instructed to press the plunger with their thumb or the pedal with their foot as soon as possible upon recognizing a red or blue circle on the image, respectively. Participants were told to ignore the location of the target on the body, and to respond only to the color of the target. This information was presented on instruction screens at the beginning of each testing phase, in white font on a black background. In addition, the procedure and instructions were explained verbally to each participant and they were given the opportunity to ask questions and clarify any uncertainties with the research assistant before beginning the task.

The computerized experimental task was employed using a custom written program on E-Prime (2.0) software, which controlled the randomized presentation of the stimuli and recorded the timing and identification of responses. Before the testing phase, each participant completed a familiarization/practice session that included 8 trials of randomized Barbie images (equal red or blue targets, on the hand or foot, and on the right or left side). Participants then individually completed the test, which lasted approximately 30 minutes in duration. Participants were offered a brief break following each block of trials.

The testing phase was comprised of 5 blocks (32 trials) of the choice response task (160 trials). The choice response task trials in each block consisted of 4 instances (one for each Barbie body type) of the 8 trials derived via the factorial combinations of target color (red, blue), location (hand, foot), and target side (right/left). Each trial began by presenting the word “READY” in large black font in the middle of a white screen for 1000 milliseconds (ms). A black fixation cross directed and maintained participants’ attention to the middle of the screen during the foreperiod. To discourage anticipations, Barbie images were presented randomly 1000-3000ms after the presentation of the fixation cross. Each image of Barbie was positioned with respect to the fixation cross, with the hand and foot roughly equidistant from the center of the cross.

Immediately following the experimental task, participants completed a self-report questionnaire for the purpose of collecting relevant demographic information. Age and ethnicity variables were used in preliminary analyses to examine between group differences in body-part compatibility effects based on age group and ethnicity. In addition, participants were asked to rate each Barbie doll (presented in an image) in terms of attractiveness, pleasantness, and desirability. For this task, the Barbie dolls were dressed identically in purple bikinis and digitally inserted onto a white background. Participants were randomly assigned to one of four orders of Barbie doll image presentations. The explicit ratings of Barbie body types were collected after the experimental task to avoid priming participants to the implicit processing portion of the study, which could have confounded automatic assessments of response time. Participants’ explicit perceptions of the original, tall, curvy and petite Barbie dolls were assessed descriptively to better understand how young girls evaluate the newly introduced body types. Upon completion of the study protocol, parents and children took part in a debriefing session. At this time, participants and their parents were provided with a more detailed description of the study purpose and hypotheses, as well as mental health resources.

### Measures

#### Self-report questionnaire

A self-report questionnaire (see S1 File) was used to collect participants’ demographic information, including age (coded as younger; 6–9 years old; older: 10–14 years old), school grade, and ethnicity (coded as the same ethnicity as Barbie: Caucasian/Russian or coded as a different ethnicity than Barbie: e.g., East Asian, South Asian, Latin American), and to assess participants’ perception and evaluation of each Barbie body type. Perceptions of Barbie were measured using ratings of each Barbie body type across three subscales, including: attractiveness (“How pretty do you think this Barbie is?”), pleasantness (“How much do you like this Barbie?”) and desirability (“How much do you want to look like this Barbie?”). Each item was assessed on a 6-point Likert-type scale, from 0 (not at all) to 5 (extremely). Participants’ overall preference for each Barbie body type was then computed as a mean score of all subscale measures.

#### Stimuli

Target stimuli were coded as body-part compatible or incompatible, with respect to the color (red/blue) and location (hand/foot) of the circle target in the Barbie image. As such, a red circle (requiring a hand response) was coded as “compatible” when presented on the Barbie’s hand, and “incompatible” when presented on the Barbie’s foot. Similarly, a blue circle (requiring a foot response) was coded as “compatible” when presented on the Barbie’s foot, and “incompatible” when presented on the Barbie’s hand.

#### Response time (RT)

In the body-part compatibility task, RT was defined as the time period from the onset of image presentation until either the thumb button or foot pedal was pressed. Prior to analysis, the RT data were screened for outliers and errors in several steps. First, response errors were identified as trials on which participants executed the wrong response (e.g., a foot, instead of a hand response, for a red stimulus). RTs on error trials were eliminated from the RT data set. Following a similar protocol used by Pacione et al. [50] for body-part compatibility task data with children, RTs shorter than 100ms were deleted as anticipation errors. Next, the data were screened for long RTs thought to represent inattention errors. Due to large inter/intra-subject variability in measures of automatic processing in children, inattention errors were identified following an initial visual scan of the raw RT data and unusually long RTs were removed from the data set (e.g., those RTs that were 1000 ms larger than the typical range of RTs for that person). Once these unusually long RTs were removed from the data set, RTs were collapsed for each condition across Barbie body types to compute mean RT values for each individual participant. As a final step, RTs greater than two standard deviations above the mean for a certain condition were considered outliers and were eliminated. In total, 5.1% of trials were deleted as response errors, and 6.8% as anticipation errors, inattention errors and statistical outliers.

After the data for these trials were removed, mean RTs for each Barbie body type under compatible and incompatible conditions were calculated for a planned comparison in the main analysis. RTs in the body-part compatibility task were assessed to evaluate how children implicitly process Barbie doll images in relation to their own body. In this way, the presence of the body-part compatibility effect (RTs on compatible trials being lower than RTs on incompatible trials) indicates a higher degree of “self-other matching” and use of cognitively-based social comparative processing when viewing the distinct Barbie body type, whereas the absence of the body-part compatibility effect (similar RTs on compatible and incompatible trials) may indicate that the image did not drive a “self-other” matching process (e.g., the viewer did not compare their own body to that of the doll in the image).

### Data analysis

An initial inspection was conducted to assess for missing data, response error, and statistical outliers. Further examinations of statistical assumptions and distributional properties (i.e., means, standard deviations, skewness and kurtosis) were then completed. Descriptive statistics were calculated for all relevant demographic and study variables, including participants’ RT data and self-reported perception of each Barbie body type. Table 1 shows mean scores of attractiveness, pleasantness, and desirability across Barbie body types, as well as overall preference (computed as a mean score of all subscale measures). Table 2 displays mean RT and response error data for each condition, across Barbie body types.

**Table 1:** Mean (M) scores and standard deviations (SD) for ratings of attractiveness, pleasantness, desirability and overall preference across Barbie body types.

	Attractiveness M (SD)	Pleasantness M (SD)	Desirability M (SD)	Overall Preference M (SD)
Original	2.11 (1.49)	1.68 (1.60)	1.53 (1.48)	1.85 (1.47)
Tall	2.16 (1.42)	1.76 (1.52)	1.45 (1.41)	1.76 (1.18)
Curvy	2.08 (1.26)	2.05 (1.45)	1.29 (1.23)	1.87 (1.10)
Petite	2.26 (1.52)	1.92 (1.48)	1.61 (1.50)	1.96 (1.36)

Note: Ratings were on a 6-point Likert-type scale from 0 to 5.

**Table 2:** Means and standard deviations of reaction times (ms) and response errors collapsed across age groups.

		Original		Tall		Curvy		Petite	
Outcome	Group	Com	Incom	Com	Incom	Com	Incom	Com	Incom
RT	6–9	889 (132.5)	953 (135.1)	943 (165.9)	939 (145.4)	934 (171.4)	968 (183.5)	904 (171.2)	951 (133.4)
	10–14	785 (162.1)	844 (175.7)	786 (175.3)	809 (159.2)	790 (162.7)	821 (143.7)	799 (176.4)	830 (162.1)
Error	6–9	0.76 (0.90)	1.29 (1.05)	0.76 (0.90)	1.24 (1.52)	0.65 (1.22)	1 (0.87)	1 (1.06)	1.41 (1.12)
	10–14	0.62 (1.36)	1.48 (1.91)	0.62 (1.36)	1.10 (1.45)	0.67 (1.15)	1.76 (1.81)	0.57 (1.16)	1.38 (1.50)

Note: Group refers to younger (6–9 years old) and older (10–14 years old) age groups. RT = reaction time, Com = body-part compatible trials, Incom = body-part incompatible trials.

Mean RTs computed from the body-part compatibility task data were submitted to a 2 (Age Group: 6–9 years old, 10–14 years old) by 4 (Barbie Body Type: original, tall, curvy, petite), by 2 (Stimulus Location: compatible, incompatible) mixed model analysis of variance (ANOVA). Age Group (younger: 6–9 years old, older: 10–14 years old) was used as a between-subjects factor, while Barbie Body Type and Stimulus Location were repeated measures factors within the ANOVA model. Mauchly’s test indicated that the assumption of sphericity was maintained across all repeated measures variables (*p* > .05). An initial model tested between group differences based on ethnicity. Mean RTs were submitted to a 2 (Ethnicity: Caucasian/Russian, Other ethnicity) by 2 (Age Group: 6–9 years old, 10–14 years old) by 4 (Barbie Body Type: original, tall, curvy, petite), by 2 (Stimulus Location: compatible, incompatible) mixed model ANOVA. For all significant effects, RT differences were further analyzed using paired samples t-tests with Benjamini-Hochberg correction. All analyses were conducted using SPSS (Version 24.0), with alpha set at 0.05.

## Results

### Participant characteristics

The sample (*N* = 38, *M*_age_ = 10, *SD* = 2.24) was split into two age groups, the younger group consisting of 17 children between the ages of 6 and 9 years old, and the older group consisting of 21 children, 10–14 years old. Participants identified as Caucasian (57.9%), East Asian (18.4%), Russian (7.8%), South Asian (5.3%), Latin American (5.3%) and as mixed / other ethnicity (5.3%). Participants came from mid-to-higher socioeconomic backgrounds, reported at least one parent who completed higher education, and resided in a large Canadian metropolitan city.

### Explicit Barbie preferences

Participants’ perceptions of Barbie varied depending on body type. Participants reported curvy Barbie as the most pleasant (or “likeable”) body type, but also as the least desirable and the least attractive. In contrast, the original and tall Barbie representations were both rated low for pleasantness, but high for desirability and attractiveness. As seen in Table 2, the petite Barbie was revealed as the most preferred body type overall.

### Body-part compatibility

A main effect was found for Stimulus Location, *F*(1, 36) = 31.39, *p* < .001, η_p_^2^ = 0.47, with RTs being shorter for compatible trials (*M* = 847ms, *SD* = 11.1) compared to incompatible trials (*M* = 883ms, *SD* = 10.8). Thus, overall, there was a body-part compatibility effect. A main effect was found for Age Group, *F*(1, 36) = 8.87, *p* < .01, η_p_^2^ = 0.198, whereby the younger age group demonstrated shorter RTs (*M* = 935ms, *SD* = 26.1) compared to the older age group (*M* = 808ms, *SD* = 21.9). There was no main effect for Barbie Body Type, *F*(3, 108) = 0.51, *p* > .05, η_p_^2^ = 0.014. Significant 2-way interaction effects were found for Barbie Body Type by Age Group, *F*(3, 108) = 2.72, *p* < .05, η_p_^2^ = 0.07, and Barbie Body Type by Stimulus Location, *F*(3, 108) = 2.84, *p* < .05, η_p_^2^ = 0.073. The 2-way (Stimulus Location by Age Group) and the 3-way (Barbie Body Type by Stimulus Location by Age Group) interactions were not statistically significant (*p*s > .05). Note that ethnicity was included as a between-subjects factor in all initial analyses. There was no significant main effect of Ethnicity and no statistically significant interactions involving Ethnicity, *p*s > 0.13, η_p_^2^ s < 0.065), and therefore, this variable was not included in the final reported analyses.

**Fig 1:**
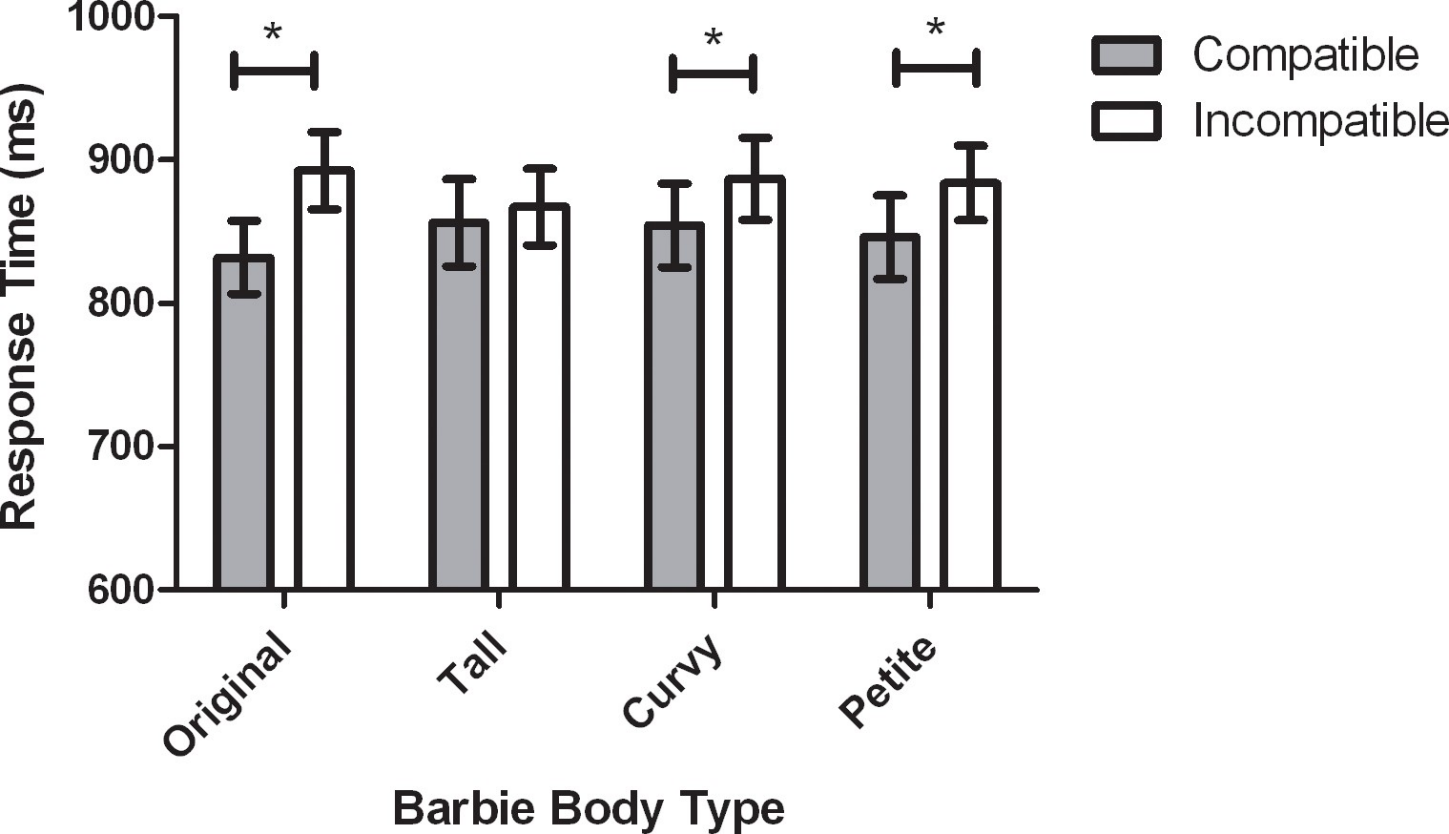
Mean RT in milliseconds for all participants as a function of Barbie body type and stimulus location. Standard error bars are included for each condition. Asterisks (*) indicate significant body-part compatibility effects (*p* < .05).

**Fig 2:**
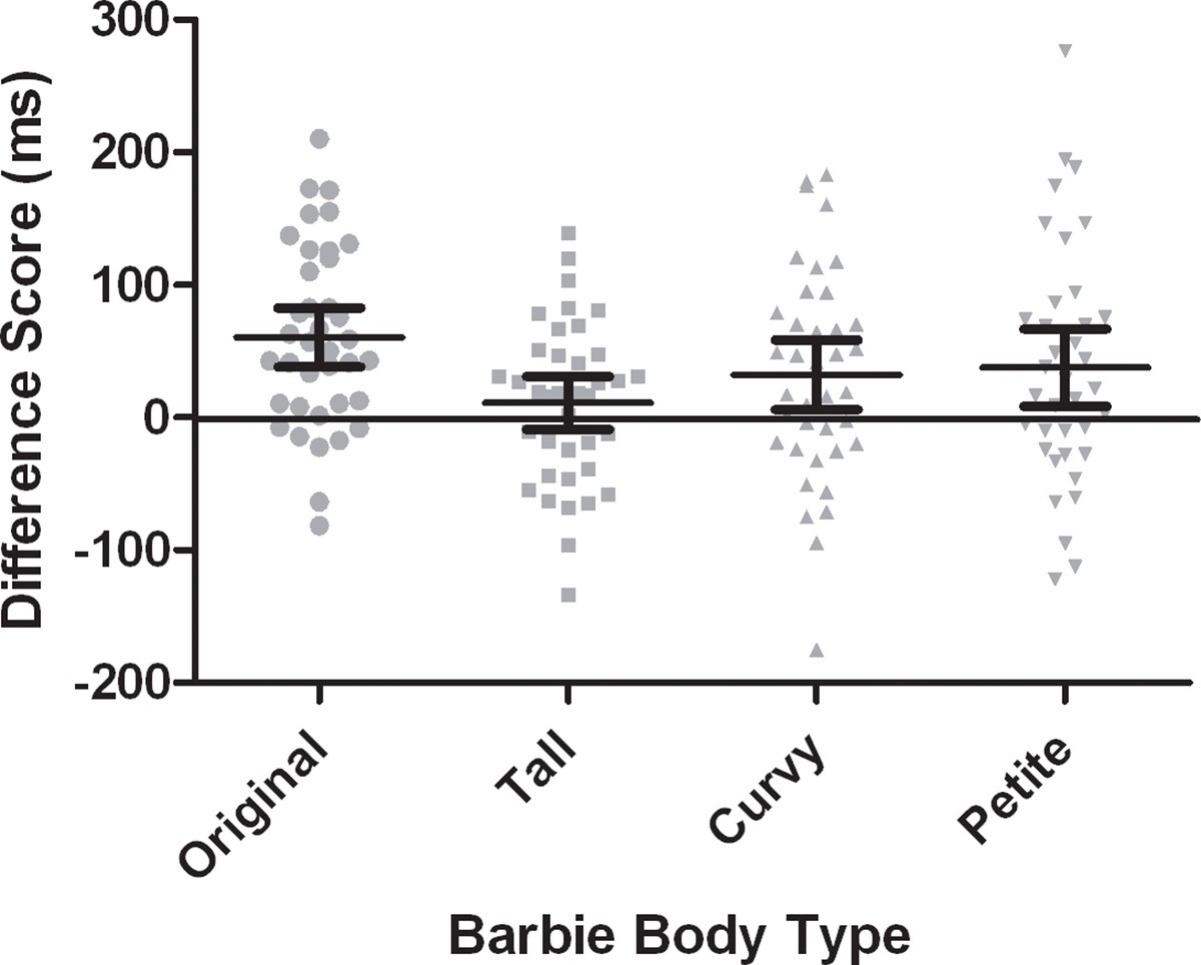
Mean differences in participants’ RT (in milliseconds) between body-part compatible and incompatible trials for each Barbie body type. 95% confidence intervals are shown for each condition.

Because the 3-way interaction was not significant, the critical interaction for the purpose of the present study was the Barbie Body Type by Stimulus Location. As such, this effect was subjected to a post hoc analysis to understand the nature of the interaction. Results from the paired samples *t*-tests with Benjamini-Hochberg corrections comparing RTs under body-part compatible and incompatible conditions across the four Barbie body types, revealed significant body-part compatibility effects for the “original” [*t*(37) = -5.48, *p* < .0125, 95% CI of the difference scores: -82.9, -38.1, *d*_*z*_ = .89], “petite” [*t*(37) = 2.63, *p* < .017, 95% CI of the difference scores: -66.8, -8.7, *d*_*z*_ = .43] and “curvy” [*t*(37) = 2.50, *p* < .025, 95% CI of the difference scores: -58.6, -6.1, *d*_*z*_ = .40], Barbie dolls. No statistically significant body-part compatibility effect was found for the “tall” Barbie [*t*(37) = -1.12, *p* > .05, 95% CI of the difference scores: -31.0, 8.9, *d*_*z*_ = .18]. Visual representations of these RT differences, depicting a Barbie-specific body-part compatibility effect, are displayed in Fig 1 and Fig 2. The non-significant 3-way interaction involving Age Group indicates that these body-part compatibility effects were not moderated by age.

## Discussion

The present novel study explored how young girls respond to the four representations of different Barbie dolls (original, tall, curvy and petite), and particularly the extent to which each body type was implicitly processed. In this way, we were able to highlight the automatic self-other matching and social comparative processes that occur when young girls view the new Barbie dolls. There were several novel findings arising from this work.

The key finding of the present study was captured in the pattern of significant body-part compatibility effects across the different Barbie dolls because this effect indexes young girls’ body representation and degree of self-other matching during exposure to Barbie. Consistent with previous findings, the body-part compatibility effect emerged overall–there was a main effect of Stimulus Location, such that RTs for body-part compatible trials were shorter compared to RTs for incompatible trials [31,35,48]. Unique to the present study was the finding that body-part compatibility effects differed depending on Barbie’s physique. These findings are contrary to the hypothesized results, where body-part compatibility effects were expected to emerge only when images of petite Barbie were observed. Alternatively, results demonstrated that automatic self-other matching processes occurred when images of petite Barbie were viewed, and also when viewing images of curvy and original Barbie. Body schema activation and implicit self-other matching did not occur upon viewing the tallest Barbie physique. These results are somewhat consistent with findings in the implicit processing of physique-salient media images among adult women [31]. In particular, model body type (e.g., “thin”, “average”, and “above average”) was found to moderate the body-part compatibility effect, demonstrating self-other matching for various physiques [31]. Based on theoretical tenets of social comparison theory [18] and self-schema theory [19], it was not expected that self-other matching processes would emerge for original and curvy Barbie. Nonetheless, the results are in line with recent empirical evidence demonstrating that young girls may partake in upward social comparisons with physiques that are considered unrealistic or not similar to themselves [10,55,56]. Indeed, research has supported that comparisons to dissimilar and/or unrealistic images of models may occur as frequently as comparisons to more relevant peers [21]. When comparing the explicit descriptive attitudes towards the Barbie images, the current results may also demonstrate support for this latter contention. Specifically, the participants reported overall higher scores for petite, original, and curvy Barbie. Taken together, these findings suggest that young girls and women process physique-salient images automatically and unconsciously, and that the body type portrayed in the image can moderate the extent to which individuals engage in self-other matching processes.

In contrast to our second hypothesis and previous studies [50], body-part compatibility effects were not moderated by age. Specifically, body-part compatibility effects in Pacione et al. [50] study were found to be age-specific, emerging only among children aged 10 years and older [50]. The present results may indicate that body-part compatibility effects are possible and measurable in young children under the age of 10, particularly when age appropriate and socially-relevant stimuli are used. This finding is important as both body schema activation and social comparative tendencies are considered automatic, cognitive processes, which develop throughout childhood and adolescence, and are influenced by interactions with peers, the media and one’s environment [24,25,36,44].

The finding that the body-part compatibility effect is moderated by the viewed body, and not by the participants’ age, may demonstrate the importance of exposure and social relevance in self-other matching processes. This possible explanation is most evident in the body-part compatibility effect observed with the original Barbie doll, which, despite demonstrating an unrealistic body type for female children and adolescents, still generated self-other matching processes among the young girls. Given that young girls have had more exposure to the original Barbie doll, this finding is less surprising. Moreover, Mattel has received positive feedback from commentators pertaining to the release of Barbie body types that promote variety and differentiation in the portrayal of body types [51]. As such, the effective self-other matching processes observed with petite and curvy Barbie may reflect that these dolls embody characteristics that the participants could likely be exposed to through interaction with peers and adults. Nonetheless, no body-part compatibility was observed with the tall body type, which emphasizes the need for future research to understand factors that may influence the nature of the relationship between body-part compatibility effects and body type. As such, the role of exposure, familiarity, play, and social interactions in shaping self-other matching processes requires further testing in samples of children and youth to explore these hypothesized explanations.

Results from the present study identify how young girls implicitly process four distinct Barbie representations, and provide a new perspective on how youth respond to physique-salient media. First, the presence of a body-part compatibility effect indicates that the body schema is activated during exposure to Barbie images, which may subsequently increase young girls’ awareness and activation of appearance-related information. According to previous research, thin ideal messages (e.g., images of ultra-thin models), when perceived as self-relevant, activate and negatively influence appearance-related self-schemas and the processing of self-related information [26–28]. Particularly, acute activation of the self-schema is thought to lead to heightened awareness of appearance related information and increased activation during subsequent exposure to physique-salient images [26,57,58]. Such increases in appearance self-schema activation has been linked to body dissatisfaction and increased weight-related anxiety among adult women [59–61]. Similar results have been reported among female adolescents between the ages of 13 and 15 years, with higher appearance self-schema activation leading to higher body-dissatisfaction [56]. While there remains a lack of experimental studies testing schema activation in children, appearance schemas have been shown to mediate the relationship between physique-salient media exposure and body dissatisfaction among 9 to 12 year old girls [62]. Therefore, original Barbie’s detrimental impact on body satisfaction [10] may be partly explained by the negative effects that physique-salient images have on body schema representations and beliefs about appearance [27,63]. This situation is concerning given the widespread popularity of the original Barbie among young girls, and the unrealistic, physique-salient beauty ideal that the doll represents.

Additionally, the pattern of body-part compatibility effects revealed in the present study are thought to indicate that female children as young as six years old may implicitly view themselves as similar and self-match with the various Barbie representations. The elicitation of an automatic self-other matching process could have implications for body image and mental health concerns in connection to physique-salient media exposure and upward appearance-based social comparisons. Indeed, previous research has shown that female youth are aware of sociocultural appearance ideals, and increasingly engage in appearance and body-related social comparisons as they mature through childhood and adolescence [64, 65]. The tendency to engage in upward appearance-based social comparisons has been associated with a host of maladaptive psychological outcomes, including increased body dissatisfaction, feelings of self-consciousness, reduced self-esteem, negative affect and unhealthy eating attitudes [21,22,66–69]. Moreover, social comparative tendencies have been positively associated with clinical indices of mental health in samples of children and adolescents, such as disordered eating symptomatology, depression, and anxiety [66,70,71]. Continuous exposure to the unrealistic body shapes of Barbie may therefore increase young girls’ vulnerability to experiencing these negative body image and mental health outcomes. However, body image and mental health concerns were not assessed in the current investigation. Given previous empirical evidence that supports the potential for negative body image and mental health outcomes associated with seeing / playing with the original Barbie [9,10], future work is needed to see if similar outcomes arise with the new Barbie dolls. Though the new body types of Barbie are more diverse than the original doll, they remain unrepresentative of the diversity of female body shapes. More research is needed to determine whether there are associations between exposure to the new Barbie dolls and clinical mental health outcomes. Considering that female children and adolescents under the age of 19 are the most prone to the negative effects of physique-salient images [63], it is imperative that research continue to focus on the automatic, cognitive nature of social comparative tendencies when young girls play with specific toys and view other physique-salient media.

### Limitations and future directions

While the present study offers insight into the cognitive indicators of body image during exposure to Barbie among young girls, several limitations should be noted. First, the convenience sample precludes generalizability. Although ethnicity was not a moderator in the present study, a larger and more diverse sample of female children would facilitate replication and the ability to investigate possible moderating factors such as ethnicity. For instance, skin tone and hair colour are important aspects of one’s cognitive body representation which may be tested in future research examining body-part compatibility effects among children and adolescents. Given Barbie’s clear representation of a young white female, it is possible that children who identify with other ethnic backgrounds will demonstrate unique body-part compatibility effects. In fact, ethnicity has been found to moderate the relationship between body-ideal internalization and body dissatisfaction, and may therefore play a protective role among girls and women [71].

Second, there are a number of variables (e.g., developmental stage, familiarity with Barbie, body satisfaction, body esteem, body-related emotions) that may influence social comparative tendencies [21,25,31], and thus may moderate the relationship between body-part compatibility effects and Barbie body type. As such, to further understand body-part compatibility effects and the implications that Barbie may have on body image, future research is needed to understand variables that moderate the strength of self-other matching processes when viewing different Barbie body types. In addition, children and youth typically display large inter/intra-subject variability in measures of automatic processing (e.g., reaction time). Thus, future research may consider controlling for attention when testing body-part compatibility effects among younger age groups. Third, the present study used a cross-sectional design and cannot infer any causal or temporal relationships between Barbie exposure and young girls’ social comparative processing of particular body types. Longitudinal studies examining the impact of each Barbie body type on appearance ideals, body-part compatibility effects and social comparison tendencies over time, as well as comparisons to other familiar dolls marketed towards children, may better inform toy distributors, parents, and teachers on the effects of these physique-salient toys. It is also recommended that future studies consider utilizing mixed-methods, especially qualitative approaches such as focus groups and interviews, to reveal a more in depth account of young girls’ experiences, opinions and preferences related to various Barbie representations.

Moreover, it is important to recognize that physique-salient products depicting gendered body ideals are marketed towards both young girls and boys [72]. While there is a large proportion of research dedicated to understanding the body image effects of physique-salient media exposure among young girls and women, more recent studies have highlighted the potential detrimental role of popular action figures which portray the muscular body ideal for young boys’ body image development [73]. Indeed, researchers suggest that male youth are at risk of endorsing the muscular body ideal and engaging in appearance-related social comparisons [21,74,75]. These findings raise concerns as body ideal internalization and increased drive for muscularity are considered risk factors for the development of eating disorders and negative health behaviours among male youth [76]. However, experimental studies examining the impact of physique-salient toys on male children and adolescents’ body image are scarce, and to our knowledge, researchers have not yet examined the automatic and implicit nature of boys’ appearance-related social comparisons. Thus, future research is needed to extend the results of the present study to young boys’ social comparative tendencies following exposure to physique-salient images and products.

In conclusion, the present study was designed to address a gap in the literature by examining how exposure to the new and diverse Barbie dolls impacts implicit processes related to how young girls perceive their bodies and engage in self-other matching. Investigating the extent to which young girls implicitly identify as similar to the Barbie body types provides new and unique evidence regarding the study of how appearance-focused toys may impact young girls’ body schema. Future research is warranted regarding the new Barbies’ impact on the body schema, body image and mental health. Findings from the present study may be used to advance theory and measurement pertaining to cognitive body image, and may inform future research which seeks to understand the impact of physique-salient dolls on social-comparative tendencies and body-image development among children and young adolescents.

## Supporting information

S1 File Self-report questionnaire [Supporting Information_CS.docx]. (DOCX)
