# Supplementary material for: Barbie’s new look: Exploring cognitive body representation among female children and adolescents
Source: PLoS One. 2019 Jun 25;14(6):e0218315. doi: 10.1371/journal.pone.0218315 (PMC6592527; doi:10.1371/journal.pone.0218315)
Supplement: S1 File — (DOCX) [file pone.0218315.s001.docx]

**Self-Report Questionnaire**

1. **Demographic Information**
2. What are your initials
3. How old are you?
4. What grade will you be going into this September?
5. What is your ethnicity?

- Caucasian (White)
- African American (Black)
- Native American
- Latin American
- East Asian (Chinese, Korean, Japanese, etc.)
- South Asian (Sri Lankan, Indian, Pakistan, etc.)
- Other (please specify)

1. **Barbie Images (Original, Tall, Curvy and Petite Barbie)**

[Image of Original Barbie was inserted here]

1. How much do you want to look like this Barbie?

Not at all Extremely

0 1 2 3 4 5

1. How pretty do you think this Barbie is?

Not at all Extremely

0 1 2 3 4 5

1. How much do you like this Barbie?

Not at all Extremely

0 1 2 3 4 5

[Image of Tall Barbie was inserted here]

1. How much do you want to look like this Barbie?

Not at all Extremely

0 1 2 3 4 5

1. How pretty do you think this Barbie is?

Not at all Extremely

0 1 2 3 4 5

1. How much do you like this Barbie?

Not at all Extremely

0 1 2 3 4 5

[Image of Curvy Barbie was inserted here]

1. How much do you want to look like this Barbie?

Not at all Extremely

0 1 2 3 4 5

1. How pretty do you think this Barbie is?

Not at all Extremely

0 1 2 3 4 5

1. How much do you like this Barbie?

Not at all Extremely

0 1 2 3 4 5

[Image of Petite Barbie was inserted here]

1. How much do you want to look like this Barbie?

Not at all Extremely

0 1 2 3 4 5

1. How pretty do you think this Barbie is?

Not at all Extremely

0 1 2 3 4 5

1. How much do you like this Barbie?

Not at all Extremely

0 1 2 3 4 5
